# Gut Microbiota in the Progression of Type 2 Diabetes and the Potential Role of Exercise: A Critical Review

**DOI:** 10.3390/life14081016

**Published:** 2024-08-15

**Authors:** Chariklia K. Deli, Ioannis G. Fatouros, Athanasios Poulios, Christina A. Liakou, Dimitrios Draganidis, Konstantinos Papanikolaou, Anastasia Rosvoglou, Athanasios Gatsas, Kalliopi Georgakouli, Panagiotis Tsimeas, Athanasios Z. Jamurtas

**Affiliations:** 1 Department of Physical Education and Sport Science, School of Physical Education, Sport Science, and Dietetics, University of Thessaly, 42100 Trikala, Greece; ifatouros@uth.gr (I.G.F.); apoulios@uth.gr (A.P.); cliakou@uth.gr (C.A.L.); ddraganidis@uth.gr (D.D.); kpapanikolaou@uth.gr (K.P.); arosvoglou@uth.gr (A.R.); athgatsas@uth.gr (A.G.); ptsimeas@uth.gr (P.T.); ajamurt@uth.gr (A.Z.J.); 2 Department of Dietetics and Nutrition, School of Physical Education, Sport Science, and Dietetics, University of Thessaly, 42100 Trikala, Greece; kgeorgakouli@uth.gr

**Keywords:** diabetes, exercise, glucose metabolism, gut microbes, pre-diabetes

## Abstract

Type 2 diabetes (T2D) is the predominant metabolic epidemic posing a major threat to global health. Growing evidence indicates that gut microbiota (GM) may critically influence the progression from normal glucose tolerance, to pre-diabetes, to T2D. On the other hand, regular exercise contributes to the prevention and/or treatment of the disease, and evidence suggests that a possible way regular exercise favorably affects T2D is by altering GM composition toward health-promoting bacteria. However, research regarding this potential effect of exercise-induced changes of GM on T2D and the associated mechanisms through which these effects are accomplished is limited. This review presents current data regarding the association of GM composition and T2D and the possible critical GM differentiation in the progression from normal glucose, to pre-diabetes, to T2D. Additionally, potential mechanisms through which GM may affect T2D are presented. The effect of exercise on GM composition and function on T2D progression is also discussed.

## 1. Introduction

Type 2 diabetes (T2D) is the predominant metabolic epidemic and a major global health risk [1]. In 2021, the International Diabetes Federation estimated that 537 million adults aged 20–79 years globally had diabetes, with almost half of them undiagnosed [1]. T2D is typically preceded by pre-diabetes (pre-D), a state of intermediate hyperglycaemia determined by impaired fasting glycaemia (IFG), impaired glucose tolerance (IGT), or both [1]. T2D and pre-D associate with life-threatening microvascular and macrovascular complications that contribute immensely to worldwide mortality and disability [2] and cause enormous medical and socioeconomic strains on countries’ healthcare systems [3]. 

T2D is related to genetic predisposition, but also obesity and inadequate physical activity [4]. Robust evidence suggests that retaining a healthy body weight, increasing physical activity and adopting a healthy diet are ways to prevent or delay the onset of T2D [4]. Additionally, growing evidence demonstrates that environmental factors such as the gut microbiota (GM) also play a crucial role in T2D development [4]. 

Regular exercise plays a vital role in the prevention and treatment of T2D [5] by improving insulin sensitivity, skeletal muscle glucose uptake, fasting blood glucose disposal, inflammatory profile, and antioxidant status [6,7,8]. Emerging evidence suggests that regular exercise may also alter the body’s GM composition in favor of health-promoting bacteria that improve tissue metabolism and insulin resistance [9], revealing another favorable influence of exercise on health. Nevertheless, research regarding the favorable effects of exercise-induced changes on GM composition in T2D and the associated mechanisms through which these effects are accomplished is still in early stages. 

This review presents the current data regarding the association of GM composition and T2D and the possible critical role of GM differences in the progression from normal glucose tolerance (NGT) to pre-D, to T2D. Additionally, potential mechanisms through which GM composition may affect T2D are presented. Finally, the potential role of exercise on GM composition and function on T2D progression is also discussed.

## 2. Pathophysiology of Type 2 Diabetes

T2D is characterized by fasting and post-prandial hyperglycemia resulting from inadequate insulin action [10]. Under physiological conditions, insulin mediates the uptake and utilization of blood glucose mainly from the skeletal muscle, liver, and adipose tissue. In T2D, although insulin levels are normal or high, these tissues become resistant to insulin, resulting in high blood glucose levels [11]. Notably, skeletal muscle is responsible for most insulin-stimulated glucose uptake and postprandial storage [12] and accounts for nearly ~90% of the whole-body glucose metabolism [13]. Thus, insulin resistance in the skeletal muscle is a crucial pathogenic defect in T2D [13]. Insulin-stimulated transportation of glucose into the skeletal muscle is primarily mediated by the translocation of glucose transporter 4 (GLUT4) to the cell membrane [14]. In the muscle of individuals with T2D, the signal cascade of insulin-stimulated glucose uptake and transport is compromised, leading to impaired muscle glycogen synthesis [13]. Insulin resistance is evident early, beginning in the pre-D stages. Hepatic insulin resistance is related to elevated endogenous glucose production by the liver, and is mainly evident in individuals with IFG. Peripheral insulin resistance is related to elevated glucose after eating or 2 h following an oral glucose tolerance test (OGTT) due to insulin resistance in skeletal muscle, and it is primarily reflected in those with IGT [15]. The decline in peripheral insulin sensitivity is likely to be caused by obesity, physical inactivity, and/or an unhealthy diet [15]. 

Glucagon is also implicated in T2D pathogenesis [16,17], and abnormal glucagon secretion contributes to hyperglycemia in both T2D [18] and pre-D [19,20,21]. Glucagon is a counter-regulatory hormone to insulin, and glucose regulation essentially depends on the coordinated secretion of glucagon and insulin from the α- and β-cells of the pancreas, respectively [17]. Under hypoglycemic conditions, glucagon is released by the pancreatic α-cells to stimulate hepatic glucose production, thus elevating circulating glucose levels. Contrarily, under hyperglycemic conditions glucagon release is suppressed and insulin release is stimulated [17]. However, in T2D fasting plasma glucagon is improperly raised and α-cell postprandial glucagon suppression is blunted or absent, resulting in high plasma glucagon concentration [16,22]. This contributes to higher endogenous glucose production by the liver and subsequently to hyperglycemia [16,18]. Additionally, β-cell dysfunction resulting in the reduced secretion of insulin contributes to the reduced restraint of hepatic glucose production in T2D [16]. Abnormal glucagon secretion is also evident in IFG and IGT [19], while reduced insulin secretion due to β-cell dysfunction results in the blunted restraint of hepatic glucose production in IFG [20]. 

Altered secretion and action of the gut hormones glucagon-like-peptide-1 (GLP-1) and glucose-dependent insulinotropic polypeptide (GIP) is another key defect in T2D [15]. These hormones act as incretin hormones, triggering insulin secretion, inhibiting glucagon secretion, inhibiting gastrointestinal motility and secretion, controlling appetite and food intake [23], and modulating energy and glucose metabolism [24]. Reduced glucose-stimulated GLP-1 responses have been reported in obese individuals and individuals with pre-D and T2D compared to individuals with NGT [25], suggesting that alterations in GLP-1 release contribute to glucose and appetite dysregulation. 

Chronic low-grade inflammation constitutes an independent risk factor for T2D onset [10,26] and is evident from the pre-D stage [27,28,29]. Potential sites of inflammation in obesity and T2D are skeletal muscle, adipocytes, the liver, and the pancreas, and interaction between these tissues seems to mediate the development of T2D [14]. Macrophage infiltration into these tissues triggers the production of pro-inflammatory cytokines [10,30,31] that lead to insulin resistance by interfering with insulin signaling in peripheral tissues [32]. Additionally, increased oxidative stress is observed in individuals with pre-D [33] and T2D [34,35]. Diminished antioxidant capacity, elevated ROS generation, and oxidation of DNA, lipids, and proteins have been detected in plasma, as well as urine and some tissues, indicative of systemic and organ-specific redox perturbations in obesity [36] and T2D [34]. Hyperglycemia-induced oxidative stress may affect the insulin signaling cascade, disturb the cellular redistribution of insulin signaling elements, decrease GLUT4 gene transcription, and alter mitochondrial activity [35].

## 3. Gut Microbiota and Type 2 Diabetes

The human gastrointestinal tract is densely populated by more than 100 trillion microorganisms, which are collectively called GM [37,38,39]. GM are classified in taxonomic units, with phyla constituting the highest level of classification, which can be subdivided into class, order, family, genus, and species [40]. *Bacteroidota* (*Bacteroidetes*) and *Bacillotta* (*Firmicutes*) are the most predominant phyla in the human GM, accounting for more than 90% of all microbes [39], followed by *Actinomycetota* (*Actinobacteria*), *Pseudomonadota* (*Proteobacteria*), *Fusobacteriota* (*Fusobacteria*), and *Verrucomicrobiota* (*Verrucomicrobia*) [41]. An individual GM profile is affected by several factors, including the host’s genetics [42], age [43], geographic location [44], race, health status, and some environmental factors such as diet [45], type of birth delivery, breastfeeding [46], antibiotics [43,45,47], antidiabetic drugs [48], and physical activity level [49,50,51]. 

The relationship between the GM and the host is complex. Under balanced situations, this relation is symbiotic. The host provides a physical niche and nutrition to the intestinal bacteria, which in turn promote human health and the body’s homeostasis. GM contribute to normal immune function [52], protect against enteropathogens [53], and regulate inflammatory responses [52] and redox status [54]. Furthermore, GM contributes to the production of short chain fatty acids (SCFAs) and vitamin synthesis [45,55] as well as the digestion and absorption of food [56], all of which relate to metabolism and energy utilization during exercise [57]. Disruptions of the normal balance between the GM and the host, namely dysbiosis, is associated with various diseases including T2D [58,59,60,61,62,63,64]. 

The first evidence linking GM and altered glucose metabolism came from studies conducted on germ-free mice that suggested that the GM may contribute to changes in glucose metabolism [65]. Subsequent human studies also indicated an association between GM and T2D [66,67,68]. As of this writing, research in this field is still in its early stages. 

Antidiabetic drugs are among the factors that may influence GM composition. Treatment with Metformin [48,69], GLP-1 receptor agonists [70], and DPP-4 inhibitors [71] have been shown to favorably alter GM composition in T2D, suggesting that antidiabetic treatment may restore the gut microbial diversity toward that seen in healthy individuals. Therefore, it has been suggested that newly diagnosed and untreated patients with T2D be distinguished from known patients with T2D under antidiabetic medication when compared to the normal population, to avoid any misinterpretation of the studies’ results [48]. However, information regarding antidiabetic medications is not always provided. In the following paragraphs of this section, only data referring to patients with T2D under no antidiabetic medication are presented, although some studies have included patients with T2D both without and under antidiabetic medication (Table 1).

Larsen et al. [67] first reported compositional modifications in the GM in humans with T2D. Specifically, the abundances of the phylum *Bacillotta* and class *Clostridia* were lower, while that of the class *Betaproteobacteria* was highly increased in patients with T2D compared to healthy controls and positively correlated with plasma glucose. Moreover, the ratios of *Bacteroidota/Bacillotta* and *Bacteroides-Prevotella/Clostridium coccoides-Eubacterium rectale*, and the proportion of *Lactobacillus*, were higher in patients with T2D and positively associated with plasma glucose. Subsequent studies also revealed differences in GM composition between normal glucose metabolism and T2D [66,68,72,73,74,75,76,77,78,79,80,85,86], and although the results vary among studies, several gut bacteria are more consistently associated with the disease [61]. 

A common finding between studies is that SCFAs-producing bacteria are decreased in individuals with T2D compared to healthy individuals [66,67,78,79]. SCFAs-producing bacteria have been positively associated with health and negatively associated with T2D [68,78]. Indicatively, *Bacteroides* abundance has been negatively associated with serum glucose [87], and *Roseburia* and *Faecalibacterium prausnitzii* may reduce inflammation and oxidative stress [88]. Lower abundances of other bacteria, mainly *Bifidobacterium* [89], *Akkermansia muciniphila* [74,78], *Sutterella* [74], and some *Clostridium* species [66] have also been reported in individuals with T2D compared to NGT individuals. These bacteria have shown beneficial effects for health, and have been negatively associated with T2D in animal [90,91,92] and human studies [74,93]. Moreover, lower abundances of *Roseburia* and *F. prausnitzii*, *Coriobacteriaceae*, some *Clostridium* species [66], and *Akkermansia* and *Sutterella* [74] were identified as highly discriminant for T2D, and have been proposed as an index for the diagnosis of T2D onset [94]. On the other hand, several pathogens belonging to the *Pseudomonadota* phylum and *Betaproteobacteria* class [67,74,78], were markedly increased in individuals with T2D compared to NGT individuals, and positively correlated with plasma glucose [67]. 

Considering the observed GM dysbiosis in patients with T2D and the association of several gut bacteria with various aspects of the disease, it seems that an altered microbiota is a feature of T2D. However, the specific bacteria that may play a causal role in T2D and the mechanisms mediating this role are yet to be clarified. Nor has a microbiome signature of T2D as yet been established. 

## 4. Mechanisms through Which Gut Microbiota May Affect Type 2 Diabetes 

**Figure 1:**
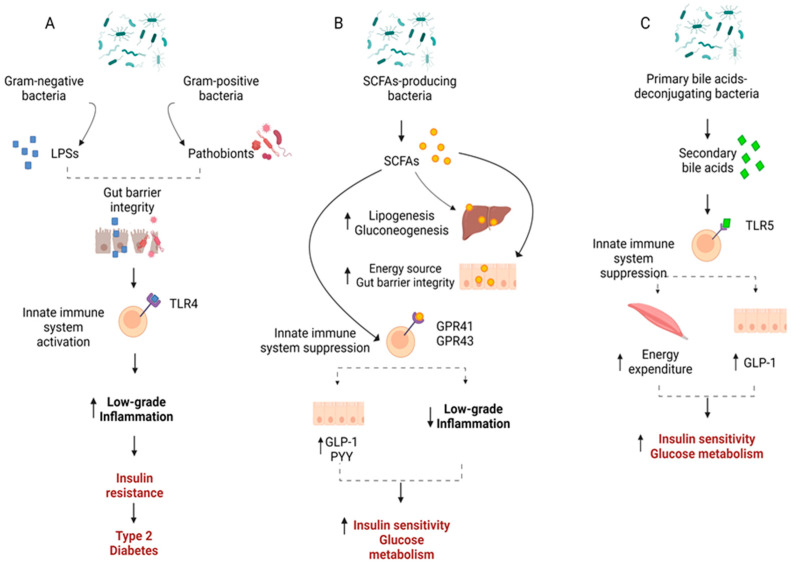
Mechanisms through which gut microbiota may affect insulin sensitivity and glucose metabolism. (**A**) Lipopolysaccharides (LPSs) of the outer membrane of Gram-negative bacteria as well as Gram-positive pathobionts activate the immune system by binding to toll-like receptor 4 (TLR4), which activates pro-inflammatory pathways and leads to low-grade inflammation, thus promoting insulin resistance and a disturbance of the glucose metabolism, and development of T2D. (**B**) Short chain fatty acids (SCFAs) produced by several bacteria improve gut barrier integrity and are used as an energy source for the gut epithelium (butyrate), substrates for gluconeogenesis and lipogenesis in the liver (acetate and propionate), or bind to G protein-coupled receptors GPR41 and GPR43, which suppress immune system activation, leading to reduced inflammation, and promote the release of glucagon-like peptide-1 (GLP-1) and peptide YY (PYY) from enteroendocrine L-cells. (**C**) Several bacteria transform prime bile acids to secondary bile acids, which bind to TLR5 and promote energy expenditure in the muscle and the release of GLP-1 from enteroendocrine L-cells. The mechanisms depicted in (**B**,**C**) result in improvement of insulin sensitivity and glucose metabolism. ↑: Increased; ↓: Decreased. Figure 1 was created with Biorender.com.

GM may positively or negatively mediate glucose regulation and insulin resistance in T2D through a number of mechanisms (Figure 1). 

Low-grade inflammation is a main driver of the insulin resistance that characterizes T2D. GM composition may affect low-grade inflammation, as some microbes may trigger pro-inflammatory responses, while others present an anti-inflammatory potential. Specifically, Gram-negative bacteria may promote inflammation through the main compounds of their outer membrane, namely lipopolysaccharides (LPSs), which provoke metabolic endotoxemia by stimulating the secretion of pro-inflammatory cytokines. Translocation of bacterial LPSs from the gut lumen into the portal circulation may lead to the activation of the innate immune system and pro-inflammatory gene expression via the TLR4/CD14 complex, TLR2, and TLR5, resulting in low-grade inflammation and consequently insulin resistance and T2D [59,95]. The elevated levels of *Pseudomonadota* and the lower taxonomic units of both *Pseudomonadota* and *Bacteroidota* found in patients with T2D compared to healthy controls [67,68,74,78] justify the link between these bacteria and low-grade inflammation. Pathobionts are also contained in Gram-positive bacteria which could further contribute to low-grade inflammation. For example, *Blautia coccoides* may stimulate the secretion of TNF-α to an even greater magnitude than the LPSs [96]. Higher relative abundances of Gram-positive pathobionts have been found in patients with T2D compared to individuals with NGT [73,78,87] and are positively associated with T2D [78]. 

On the other hand, higher levels of *Bifidobacterium* provoked by prebiotic feeding in obese mice reduced endotoxemia, inflammation, and oxidative stress [97]. Additionally, supplementation with *Bifidobacterium pseudocatenulatum* reduced LPSs-producing Pseudomonadota, reduced inflammation, and improved insulin sensitivity and glucose regulation [98], while *Bifidobacterium lactis* supplementation improved insulin-dependent glucose uptake and GLUT4 transportation, increased the activation of the glycogen synthesis associated pp-1 gene, and decreased the activation of the hepatic gluconeogenesis-regulated genes PCK1 and G6PC [99]. Similarly, *Roseburia intestinalis* supplementation induced an anti-inflammatory pattern by increasing IL-22 and reducing INFγ and IL-17 in mice [100]. *Roseburia intestinalis* supplementation also inhibited LPS-induced secretion of IL-17 in an in vitro model of a human colon epithelial cell line [101]. *Lactobacillus casei* and *Lactobacillus paracasei* supplementation reduced circulated LPSs, IL-1β, and IL-8, and improved glucose tolerance and HbA1c in T2D rats [102]. *L. paracasei* was also found to down-regulate the LPS-induced generation of TNF-α and IL-1β by monocyte-macrophages, and to induce the expression of inhibitors of NF-Κb [99]. *A. muciniphila* supplementation reduced LPSs, TNF-α, and MDA levels, improved body composition, reversed hyperglycemia, and reduced insulin resistance in animals [90,92,93]. 

Considering the potent activation of the innate immune system by the translocation of GM LPSs from the gut lumen into the portal vein, it is obvious that gut barrier integrity is crucial for the preservation of intestinal homeostasis and metabolism [103]. However, dysbiosis in GM may result in the destruction of the intestinal barrier and increased gut permeability, which facilitates the translocation of metabolites and pathogens from the intestines into the circulation and tissues, thus triggering systemic immune alterations and low-grade inflammation [59,97] in obesity and T2D. Contrarily, the restoration of the GM with specific beneficial genera has been associated with the enforcement of the gut barrier. *A. muciniphila* plays a key role in maintaining the integrity of the mucin layer, reducing inflammation, and protecting against obesity and T2DM [90]. Additionally, prebiotic and probiotic supplementation induced increases in *A. muciniphila* [90,104], *Bifidobacterium* [97], *Bacteroides vulgatus, Bacteroides dorei* [105], *F. prausnitzii* [106], *L. casei,* and *L. paracasei* [102]. Prebiotic and probiotic supplementation also improved gut barrier integrity, tight junction assembly, and LPSs-induced gut permeability. 

GM may also affect the glucose metabolism in T2D via the production of SCFAs. The main SCFAs butyrate, acetate, and propionate are metabolites produced via the fermentation of complex and non-digestible polysaccharides by the colonic GM, and they are either used by enterocytes or transported into the circulation across the gut epithelium [107]. Bacteria of the *Bacillotta* phylum mostly produce butyrate, while those of the *Bacteroidota* phylum mostly produce acetate and propionate [108]. SCFAs may exert their influence on glucose homeostasis through several mechanisms. SCFAs modify the metabolism of carbohydrates, lipids, and proteins both in vitro and in skeletal muscle tissues, increase the retention of skeletal muscle mass, insulin sensitivity, and blood flow, and reserve an oxidative phenotype [109]. SCFAs also bind to the G protein-coupled receptors GPR41 and GPR43 and promote the release of glucagon-like peptide-1 (GLP-1) and peptide YY (PYY) from enteroendocrine L-cells [110,111,112]. GLP-1 acts as an incretin hormone, triggering insulin secretion, inhibiting the secretion of glucagon and gastrointestinal motility and secretion, and controlling appetite and food intake [23], while PYY is a key modulator of energy and glucose metabolism [24]. Propionate supplementation may prevent weight gain in overweight humans [110], thus preventing the induction of obesity and T2D development. Butyrate and propionate may also influence glucose metabolism by triggering intestinal gluconeogenesis, which may reduce hepatic glucose production [113]. Additionally, butyrate maintains gut barrier integrity by inducing mucin synthesis [114], reducing paracellular permeability through PPAR-γ pathways [115], and regulating the assembly of tight junctions through the activation of AMPK [116]. SCFAs may also act as anti-inflammatory mediators and regulate inflammation [117]. Acetate has been reported to suppress inflammation through GPR43 signaling in immune cells [118], and butyrate to decrease LPSs-induced expression of pro-inflammatory cytokines via inhibition of NF-κB and IκBα degradation [117]. The above favorable properties of SCFAs suggest that a GM composition including SCFAs-producing bacteria could alleviate T2D. 

Another route through which GM may affect glucose homeostasis is the manipulation of bile acids, which act as signaling molecules [59]. GM can transform primary bile acids to secondary bile acids. Primary bile acids regulate bile acid and lipid and glucose metabolism via activation of the nuclear farnesoid X receptor (FXR), while secondary bile acids activate the G protein-coupled receptor 5 (TGR5), which triggers GLP-1 secretion from the enteroendocrine L-cells and increases energy expenditure in muscles [119]. Both mechanisms lead to improved insulin sensitivity and glucose tolerance. Under GM dysbiosis the above pathways may be compromised. Interestingly, the increase of Gram-negative bacteria (mostly *Pseudomonadota*) and the reduction of the beneficial Gram-positive bacteria (mostly *Bacillotta*) induced by antibiotics treatment led to a deterioration of insulin sensitivity along with a reduction in secondary bile acids [120]. Furthermore, changes in fecal bile acid concentrations were primarily correlated with alterations of the abundance of *Bacillotta* [120]. These data indicate that GM, especially those classified in *Bacillotta,* may support bile acid and glucose metabolism in humans.

Considering the above mechanisms by which GM may affect T2D, the manipulation of GM toward a more favorable composition and function regarding these mechanisms would be of great importance for preventing and/or improving T2D.

## 5. Gut Microbiota in the Progression from Normal, to Pre-Diabetes, to Type 2 Diabetes

Only a few studies have addressed the differences in GM composition that may characterize the transition from NGT to pre-D to T2D (Table 2).

Similarly to T2D, the reduction of SCFAs-producing bacteria is evident from the pre-D stage. The abundance of *Ruminococcaceae* [122], *Roseburia* [78,80,82], *F. prausnitzii* [60,82,85,121,123], *Clostridium,* and *Eubacteriales* [80,85,121,122] decreased from NGT to pre-D, with *Roseburia* abundance further decreasing from pre-D to T2D [78]. Also consistent between the studies is the decrease in the abundances of other health-promoting bacteria and the increase in the abundances of Gram-negative and/or pathogens-containing taxa from NGT to pre-D to T2D. More specifically, the abundance of the *Verrucomicrobiota phylum* [73,78] and *A. muciniphila* (belonging to *Verrucomicrobiota*) reduced from NGT to pre-D [74,78,121,123]. *A. muciniphila* [80] and *Bifidobacterium* [123] further decreased from pre-D to T2D. On the other hand, the abundances of the Gram-negative *Escherichia coli* [80,83,123], *Haemophilus* [83,122], *Sutterella* [72,78,121], *Serratia* [73], and *Betaproteobacteria* [78] increased from NGT to pre-D, and even more in the transition from pre-D to T2D. All of the above bacterial taxa belong to the *Pseudomonadota* phylum, which has also been found to increase from NGT to pre-D [80,82,83]. The abundances of other Gram-negative bacteria, that is, *Megasphaera* [80,83] and *Megamonas* [83,122], also increased from NGT to pre-D. These bacteria belong to the class *Negativutes,* which also increased from NGT to pre-D and further from pre-D to T2D [83]. Additionally, lower *A. muciniphila* [74,80] and *F. prausnitzii* [80] and higher *Sutterella* [74] and *E. coli* [80] abundances were identified as highly discriminative factors for the progression from NGT, to pre-D, to T2D.

Interestingly, there is evidence that the GM of healthy individuals who are going to develop T2D is already altered from the normoglycemic stage. Wang et al. [124], examined the GM of individuals with NGT whose glycemic stage was reexamined after a 4-year follow-up period. The baseline GM of those who developed T2D had decreased abundances of *Bifidobacterium longum*, *Coprobacillus* unclassified, and *Veillonella dispar*, and increased abundances of *Roseburia hominis*, *Porphyromonas bennonis*, and *Paraprevotella* unclassified compared with their controls. Furthermore, baseline *B. longum* was negatively correlated with fasting and 2-h glucose at the follow-up, suggesting that healthy individuals with depleted *Bifidobacterium* species may be more prone to developing T2D later in life.

It is noteworthy that the metabolic abnormalities differ between IFG, IGT, or both IFG and IGT at pre-D stages, and may result in different T2D phenotypes [19]. However, only two studies have completely characterized the pre-D stages and addressed the differentiation of GM in the progression from NGT to IFG or IGT+IFG in treatment-naïve individuals and individuals with T2D [84,85]. Wu et al. [85] reported more pronounced reductions of butyrate-producing GM and increased opportunistic pathogens than are found in individuals with NGT in individuals with IGT, IGT+IFG, and T2D, independent of age, sex, and BMI. Additionally, Diener et al. [84] identified increasing *Escherichia* and *Veillonella*, with decreasing *Blautia* and *Anaerostipes* abundances, as strong markers for the disease’s progression. More research is warranted to clarify whether different pre-D metabolic characteristics also relate to different GM compositions.

Obesity and diet may predispose an individual to T2D [4] and should be considered when examining GM composition in the progression of glucose intolerance. However, these parameters are not usually addressed. In fact, none of the above studies addressed the effect of different BMI on GM through the progression of glucose tolerance stages. Nevertheless, in most of the studies, individuals with NGT were already overweight before the development of pre-D [72,73,76,80,83,84,121,122]. The association of GM with BMI in individuals with pre-D [120,121], alongside the early evidence for GM composition altered from the normoglycemic status in those who develop T2D [124], indicates that excess body fat may contribute to the observed GM changes in the progression from NGT to pre-D to T2D. On the other hand, a confounder analysis showed that the associations between identified bacterial genera and obesity-related clinical variables (BMI and visceral fat) are lost when correcting for glucose intolerance phenotypes; diabetes measures explained most of the associations between bacterial abundances and obesity but not vice versa [84]. Thus, different phenotypes of glucose intolerance may be considered when estimating the association between GM and obesity in the development of T2D.

Regarding diet, different dietary patterns may result in different GM compositions. For example, high-carbohydrate diets are associated with a predominance of *Prevotella* while high-fat/protein diets are associated with a higher abundance of *Bacteroides* [125]. Similar findings have been reported for individuals with different glucose tolerances. Egshatyan et al. [73] reported higher *Bacteroides* and lower *Prevotella* representation as well as higher incidence of pre-D and T2D in individuals consuming a high-fat/protein diet compared to those consuming a high-carbohydrate diet. Notably, the association of some bacteria with glucose intolerance may remain even after correcting for dietary patterns [73]. *Blautia* and *Serratia* abundances remained higher, while *Verrucomicrobia* abundance remained lower in patients with pre-D and T2D even when they consumed fewer carbohydrates and fat, or an amount of carbohydrates and calories equal to that consumed by individuals with NGT [73]. This could mean that the association of some bacteria with glucose intolerance is beyond dietary patterns. The different phenotypes of glucose intolerance may also be considered when estimating the association between GM and diet in the development of T2D.

Considering the above, GM composition differentiates between the three stages of T2D progression, with dysbiosis being evident from the pre-D stage. The abundance of some microbes seems to be more consistently related to the pre-D stage and could be used as a potential marker for a high risk of diabetes. However, this has yet to be determined.

## 6. The Potential Role of Exercise on Gut Microbiota in Pre-Diabetes and Type 2 Diabetes

Regular physical exercise plays an important role in the prevention and treatment of T2D [5,126,127]. Briefly, regular exercise improves body composition, energy balance [128], fasting blood glucose [6], HbA1c [129], insulin sensitivity [6], skeletal muscle glucose uptake [7], inflammation [130], and antioxidant defenses [36,131]. Another emerging role of regular exercise on health is its potential to favorably alter the GM composition and function toward health-promoting bacteria [9,132,133]. However, most of the evidence in this area comes from studies in animals. Human studies are limited, not only of individuals with pre-D and T2D, but also of healthy individuals. Nevertheless, greater microbial diversity was seen in athletes and physically active individuals compared with sedentary individuals [49,50,51], and microbial diversity was associated with cardiorespiratory fitness in healthy adults [134]. Additionally, higher proportions of *Bacillotta* to *Bacteroidota*, and/or higher proportions of the health-promoting bacteria *A. muciniphila*, *Bifidobacterium*, and the SCFAs-producing *Faecalibacterium* and *Roseburia*, were reported in elite athletes [49,51] and physically active individuals [50,134] compared to sedentary controls. These exercise-induced changes in GM are usually associated with better metabolic health [135]. Thus, it is possible that the benefits of exercise in patients with T2D are partly mediated by exercise’s effects on the GM. This notion is further supported by the potential of several gut bacteria to trigger anti-inflammatory pathways and promote INS-signaling pathways in all relevant tissues [49]. Thus, exercise-induced changes in GM composition seem to be another way by which regular exercise confers health-promoting effects in T2D. However, as far as we know, only five studies have addressed these potential exercise effects in individuals with impaired glucose regulation, of which one was conducted on mice and four on humans (Table 3).

In the animal study, exercise exerted independent effects on the GM of animals with T2D and NGT [136]. Six weeks of treadmill running resulted in higher abundances of *Bacillotta* spp. and a lower ratio of *Bacteroides/Prevotella* spp. in both diabetic mice and trained mice with NGT compared to their sedentary littermates. Interestingly, exercise increased the abundance of *Bifidobacterium* spp. in mice with NGT, but decreased it in mice with T2D. The authors suggested that exercise training may favorably affect *Bifidobacterium* levels in NGT but may not be equally effective in the metabolically challenged states of T2D. In the same study [135], exercise increased *Clostridium cluster I* in mice with both NGT and T2D. Moreover, T2D was independently related to a higher abundance of *Clostridium cluster XI*, while exercise correlated with a lower *Bacteroides/Prevotella* spp. ratio and abundance of *Methanobrevibacter* spp., and higher abundances of *Lactobacillus* spp. and the butyrate-producer *Clostridium leptum*. However, this study did not report baseline measurements and was limited in reporting differences in GM only at the end of the training period.

Low-grade inflammation and intestinal permeability are two of the main potential mechanisms through which the GM may mediate T2D, and the limited human studies have shown that exercise training may attenuate both through alterations of the gut flora composition. Pasini et al. [137] reported that six months of combined endurance, resistance, and flexibility training, although it did not modify the concentrations of gut bacteria, attenuated gut flora dysbiosis by reducing the massive concentration of *Mycetes* and *Candida albicans*. These changes were accompanied by a reduction of CRP and zonulin concentrations and the improvement of other T2D-related health outcomes. Additionally, zonulin and CRP levels correlated with the amount of *Mycetes*, and the authors suggested a link between gut *Mycetes*, intestinal barrier function, and consequent systemic inflammation, influencing the glucose metabolism in T2D [137].

Even a relatively short period of exercise training may confer GM adaptations in patients with pre-D and T2D. Motiani et al. [138] reported alterations in GM, alongside an attenuation of intestinal inflammation and metabolic endotoxemia and an improved glycemic profile, after 2-weeks of sprint interval training (SIT) or moderate intensity continuous training (MICT). These changes were depicted by the reduction of TNF-α and the intestinal inflammatory marker lipopolysaccharides-binding protein (LBP), alongside an improvement of HbA1c, a reduction of the whole-body and abdominal fat mass, and a decrease in *Bacillotta/Bacteroidota* ratio and the abundances of *Blautia* and *Clostridium*. Additionally, baseline insulin-stimulated colonic glucose uptake associated inversely with the abundance of *Blautia*, *Bacillotta*, and the *Bacillotta/Bacteroidota* ratio, and positively with the abundance of *Bacteroidota*. Lower abundances of *Blautia* were also related to improved whole-body insulin sensitivity. These correlations suggest a link between GM, intestinal substrate uptake, and whole-body metabolism [138]. 

Different exercise intensities seem to increase different health-promoting bacteria and metabolic pathways in patients with T2D. In the study of Torquati et al. [139] the implementation of 8 weeks of moderate intensity combined resistance and MICT increased the relative abundances of *Bifidobacterium*, *A. municiphila*, and the butyrate-producers *Lachnospira eligens*, *Enterococcus* spp., and *Clostridium Cluster IV*, while high intensity combined resistance and HIIT increased the abundances of other butyrate-producers from *Eryspelothrichales* and *Oscillospirales* and the methane-producer *Methanobrevibacter smithii.* Additionally, MICT resulted in higher pyruvate metabolism while HIIT resulted in reduced bacterial pathogenic pathways.

Despite the established improvement of T2D with regular exercise, it has also been reported that a great percentage of individuals, ranging from 7% to 63%, do not respond with regards to insulin sensitivity and glucose homeostasis [141]. Interestingly, GM has also been reported to be differentially affected in pre-D responders and non-responders to exercise. Liu et al. [140] reported that exercise training for 12 weeks remarkably improved fasting insulin, HOMA-IR, and insulin sensitivity in responders, while no obvious changes were recorded in non-responders. The heterogeneous changes on the above clinical indices between responders and non-responders were also related to differential changes in the GM. Compared to responders, the GM of non-responders after training was similar with that of the sedentary groups, suggesting a maladaptation of the GM. In responders, *Bacteroides xylanisolvens*, *Alistipes putredinis,* and inflammation-associated *Alistipes shahii* decreased and *Streptococcus mitis* and *Lanchospiraceae* increased, while in non-responders, *A. shahii* increased and *Ruminococcus gnavus* decreased after training. The microbiomes of responders also showed an enhanced capacity for SCFAs biosynthesis and branched-chain amino acids (BCAAs) catabolism, while those of non-responders showed an enhanced capacity for the production of detrimental metabolites and substrates for oxidative stress and inflammation. Distinct alterations of GM metabolites between responders and non-responders after training were also evident. Fecal abundances of propionate and GABA increased in responders, while glutamate increased and GABA decreased in non-responders. The differential alterations of these microbial metabolites may underlie the distinct metabolic responses to exercise intervention in responders and non-responders. Furthermore, transplantation of fecal microbiota from responders, but not from non-responders, mimicked the effects of exercise on microbial species and their metabolites as well as on glucose homeostasis and insulin resistance in obese mice [140].

**Figure 2:**
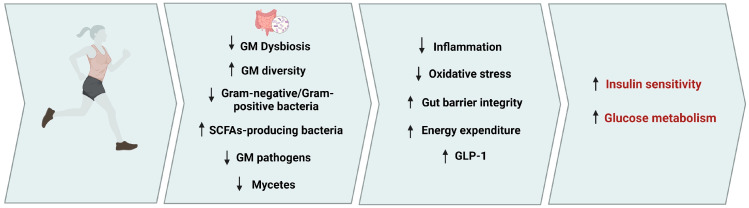
The effect of exercise-induced changes of gut microbiota in type 2 diabetes. Regular exercise may correct a dysbiosis of the GM by increasing the diversity and the abundances of health-promoting bacteria and metabolites and decreasing GM pathogens. These beneficial changes in GM composition are accompanied by a reduction of systemic inflammation and oxidative stress and an increase of gut barrier integrity and energy expenditure and incretin function, which in turn may improve insulin sensitivity and glucose metabolism. ↓: Decreased; ↑: Increased. Figure 2 was created with Biorender.com.

The above findings, although still limited, indicate that regular exercise of several modes may lead to beneficial changes in the GM related to T2D. Furthermore, the benefits of regular exercise in T2D may be maximized by targeting the GM from earlier stages, before the development of the disease. Figure 2 illustrates possible mechanisms through which regular exercise may mediate the prevention and/or amelioration of T2D.

## 7. Conclusions

Growing evidence suggests that GM dysbiosis is associated with T2D pathophysiology, manifesting in the stage of pre-D. Different microbes are related to different signaling pathways that may affect the host’s metabolism and either prevent the development of, or create a predisposition to T2D. Unraveling dysbiosis early, from the pre-D stage or even earlier, and determining a GM signature related to the risk of developing the disease, would be of great importance to create strategies to modulate the GM composition toward specific bacteria and prevent or delay the progression of the disease. Emerging evidence suggests that regular exercise may comprise such a strategy. The benefits of exercise in T2D may be maximized by targeting the GM from the NGT and pre-D stages before the development of the disease. However, current data are limited, and more research is warranted to establish a GM signature through the progression from NGT to pre-D and ultimately to T2D and to elucidate the effect of exercise-induced GM changes on this progression, as well as the mechanisms through which these effects are accomplished. Future studies should aim to reveal the most effective exercise characteristics for optimal GM adaptations for the prevention and/or improvement of T2D via individualized exercise prescriptions.

## Figures and Tables

**Table 1:** Gut microbiota composition and function in T2D patients compared to healthy individuals.

Study	Gut Microbiota Composition	Gut Microbiota Function	Associations of GM with T2D
**Karlsson 2013 [66]**	**Species model** ↑ 4 *Lactobacillus* species ↓ 5 *Clostridium* species **Metagenomic clusters (MGCs) model** ↑ *Eubacteriales*, 2 *Clostridium clostridioforme*, *Lactobacillus gasseri*, *Streptococcus mutans* ↓ *Roseburia*, 2 unknown *Clostridium*, 5 *Eubacteriales*, *Eubacterium eligens*, *Coriobacteriaceae*, *Bacteroides intestinalis*	↑ pathways for starch and glucose metabolism, fructose and mannose metabolism, ABC transporters for amino acids, ions and simple sugars, glycerolipid metabolism and fatty acids biosynthesis, cysteine and methionine metabolism ↓ pathways for flagellar assembly and riboflavin metabolism	**Positive associations** *Lactobacillus* species with FPG, HbA1c *Clostridium* species with adiponectin, HDL-C **Negative associations** *Clostridium species* with fasting GLU, HbA1c, INS, C-peptide, TG *B. intestinalis* with insulin, WC
**Larsen 2010 [67]**	↑ *Betaproteobacteria*, *Bacilli* ↓ *Bacillotta*, *Clostridia*		**Positive associations** Bacteroidota/Bacillotta with GLU tolerance (OGTT) Betaproteobacteria, Bacteroides-Prevotella/Clostridium coccoides-Eubacterium rectale with FPG
**Qin 2012 [68]**	↑ *Bacteroides caccae*, *Clostridium hathewayi*, *Clostridium ramosum*, *Clostridium symbiosum*, *Eggerthella lenta*, *Escherichia coli*, *Akkermansia muciniphila*, *Desulfovibrio* sp. ↓ *Eubacteriales* sp. *SS3/4*, *E. rectale*, *Faecalibacterium prausnitzii*, *Roseburia intestinalis*, *Roseburia inulinivorans*, *Haemophilus parainfluenzae*	↑ pathways for membrane transport of sugars, branched-chain amino acid transport, methane metabolism, xenobiotics degradation and metabolism, sulfate reduction, oxidative stress resistance, drug resistance ↓ pathways for bacterial chemotaxis, flagellar assembly, butyrate biosynthesis, metabolism of cofactors and vitamins	
**Chavez-Carbajal 2020 [72]**	↓ Richness, ≠ β-diversity ↑ *Bacteroidota*, *Sutterella* ↓ *Bacillotta* **Community drivers** *Sutterella*, *Prevotella*, *Ruminococcus*		**Positive associations** *Enterococcaseae* with age & body fat *Prevotella* with gender *Dorea* with physical activity **Negative associations** *Kaistobacter* with lipid intake *Erwinia* with SBP *Fusobacterium* with weight
**Egshatyan 2016 [73]**	↑ *Blautia*, *Serratia* ↓ *Verrucomicrobiota*		**Positive associations** Prevotella with high CHO-T consumption Bifidobacterium with high starches consumption Verrucomicrobiota with GLU tolerance (OGTT) Blautia and Serratia with GLU intolerance (OGTT) **Negative associations** *Catenibacterium* genus with high sugar consumption *Blautia* with high starches consumption *Bifidobacterium* with high-calorie food, high-cholesterol and ethanol consumption
**Gaike 2020 [74]**	↓ α-diversity, ≠ β-diversity ↑ *Bacillotta/Bacteroidota*, *Bacillotta*, *Pseudomonadota*, *Lactobacillus* ↓ *Bacteroidota*, *Verrucomicrobiota*, *Akkermansia*, *Blautia*, *Prevotella*, *Ruminococcus* **Community drivers** *Sutterella*, *Prevotella*, *Ruminococcus*		**Positive associations** *Prevotella* with histidine *Akkermansia* with histidine, total antioxidants *Blautia* with histidine, total antioxidants *Ruminococcus* with histidine *Megasphaera* with FPG, HbA1c *Escherichia* with FPG, tyrosine, lipid peroxides *Lactobacillus* with FPG, HbA1c, isoleucine *Sutterella* with HDL-C *Acidaminococcus* with FPG, VLDL-C, leucine, isoleucine, tyrosine, and lipid peroxides **Negative associations** *Akkermansia* with FPG, HbA1c, lipid peroxides, leucine, tryptophan *Sutterella* with FPG, histidine
**Inoue 2017 [75]**	↓ *Blautia*	↑ Pathways for glycolysis/gluconeogenesis, tyrosine metabolism, naphthalene degradation, insulin signaling, phenylalanine metabolism, butirosin and neomycin biosynthesis, drug metabolism-cytochrome P450, metabolism of xenobiotics by cytochrome P450, retinol metabolism, ethylbenzene degradation, proximal tubule bicarbonate reclamation ↓ pathway for oxidative phosphorylation	**Negative associations** *Blautia* with HbA1c
**Lambeth 2015 [76]**	↑ *Collinsella*, *Enterobacteriaceae* unknown genus		
**Sedighi 2017 [77]**	↑ *Lactobacillus*, *Fusobacterium* ↓ *Bifidobacterium*		
**Zhang 2013 [78]**	↓ α-diversity ↑ *Bacillotta*, *Betaproteobacteria*, *Clostridia*, *Lachnospiraceae*, *Ruminococcus*, *Dorea*, *Prevotella*, *Collinsella*, *Subdoligranulum*, *Eubacterium*, *Sporobacter*, *Abiotrophia*, *Peptostreptococcus*, *Clostridialles*, *Clostridialles* sp. SS3/4 ↓ *Bacteroides*, *Streptococcus*, *F. prausnitzii*, *Haemophilus parainfluenzae*, *Roseburia*, *Megamonas*		**Positive associations** GM composition with FPG and CRP **Negative associations** α-diversity with fasting INS
**Zhao 2019 [79]**	↑ *Bacillotta*, *Pseudomonadota*, *Eubacterium hallii*, *Eubacterium ventriosum*, *Ruminococcus torques*, *Blautia*, *Coprococcus*, *Lachnospiraceae*, *Sabdoligranulum*, *Dialister* ↓ *Bacteroidota*, *Bacteroides*, *Prevotella*, *Ruminococcus 2 * **Fecal Metabolites** ↓ Isobutyrate, hexanoic acid, cholic acid, oleanolic acid ↑ Linolenic acid, diacylglycerol	↓ pathways for tricarboxylic acid cycle, sugar metabolism ↑ pathways for glycerophospholipid metabolism, synthesis and degradation of ketone bodies, fatty acid metabolism	**Positive associations** Microbial modules (MM) 35 (*Prevotella*) and MM19 (*Streptococcus, Weissella, Pseudobutyrivibrio, Veillonella*) with BMI, WC, WHR, DBP, SBP, TG, CHOL-T, HDL-C LDL-C, lysophosphatidylcholine, linolenic acid MM12 (*Akkermansia)* with FPG, HbA1c, linolenic acid, lysophosphatidylcholine MM15 *(Lachnospiraceae*) and MM16 (*Ruminococcaceae*) with lysophosphatidylcholine **Negative associations** MM32 (*Lachnospiraceae*, *Blautia*, *Marvinbryantia*, *Blautia)* with T2D, DBP, SBP, CHOL-T, TG and HDL-C MM15, MM16 with acetate
**Zong 2019 [80]**	↑ *B. caccae*, *Bacteroides finegoldii*, *Collinsella intestinalis*, *Megasphaera elsdenii* ↓ *Dialister invisus*, *Roseburia hominis*, *E. coli*, *A. muciniphila*, *Clostridium bartlettii*	↑ Sugar phosphotransferase systems, ATP-binding cassette transporters of amino acids, bacterial secretion system	
**Wang 2017 [81]**	↑*Veillonellaceae * ↓ *Erysipelotrichaceae*		
**Gravdal 2023 [82]**	↑ *Dorea*, *Dorea formicigenerans*, *Dorea longicatena*, *Erysipelotrichia*, *Erysipelotrichales*, *Erysipelotrichaceae*, *Turicibacter*, *Turicibacter sanguinis* ↓ *Anaerotignum*		
**Zhang 2021 [83]**	↑ *Negativicutes*, *Finegoldia*, *Megasphaera*, *Prevotella*, *Alloprevotella* ↓ *Bacteroides*, *Paraprevotella*, *Helicobacter*		
**Diener 2020 [84]**	↑ *Escherichia/Shigella*, *Veillonella* ↓ *Anaerostipes*, *Blautia*		**Positive associations** *Escherichia* with HbA1c and insulin sensitivity *Veillonella* with CRP *Ruminococcaceae* with α-diversity **Negative associations** Escherichia, Veillonella, Fusobacterium, Flavonifractor, Parasutterella with α-diversity Anaerostipes, Blautia with IL-6

**Note**: Only the statistically significant results referring to T2D patients without antidiabetic or other treatment are presented. ↑: Increased; ↓: Decreased; GM: Gut microbiota; T2D: Type 2 diabetes. FPG: Fasting plasma glucose; HbA1c: Glycated hemoglobin; HDL-C: High-density lipoprotein; GLU: Glucose; INS: Insulin; TG: Triglycerides; WC: Waist circumference; OGTT: Oral glucose tolerance test; SBP: Systolic blood pressure; CHOL-T: Total cholesterol; VLDL-C: Very low-density lipoprotein; CRP: C-reactive protein; BMI: Body mass index; WHR: Waist to hip ratio; DBP: Diastolic blood pressure; LDL-C: Low-density lipoprotein; IL-6: Interleukin 6; α-diversity: the mean species diversity in sites or habitats at a local scale; β-diversity: the ratio between regional and local species diversity; richness: a simple count of species.

**Table 2:** Gut microbiota composition and function in the progression from NGT to pre-D, to T2D.

Study	From NGT to Pre-D	From Pre-D to T2D
**Chavez-Carbajal 2020 [72]**		↓ Richness ↑*Sutterella* ↓ β-diversity
**Gaike 2020 [74]**	**Community drivers** *Blautia*, *Sutterella*	↑ *Bacillotta/Bacteroidota*
**Inoue 2017 [75]**	↑ *Blautia*, *Serratia * ↑ *Prevotella * ↓ *Verrucomicrobiota*	↑ *Blautia*, *Serratia*
**Lambeth 2015 [76]**	↑ *Chloracidobacteria*, *Pseudonocardiaceae* unknown genus	↑ *Collinsella*, unknown genus of *Enterobacteriaceae* ↓ *Chloracidobacteria*, *Pseudonocardiaceae* unknown genus
**Zhang 2013 [78]**	↑ *Betaproteobacteria*, *Prevotella*, *Megamonas*, Eubacteriales sp. SS3/4 ↓ *Verrucomicrobiota*, *Verrucomicrobiia*, *Roseburia*, *A. muciniphila*, *Streptococcus*	↑ *Betaproteobacteria*, *Sutterella*, *Collinsella*, Clostridia, Subdoligranulum, Clostridialles sp. SS3/4 ↓ *Bacteroides*, *Streptococcus*, *Roseburia*
**Zong 2019 [80]**	↑ *E. coli*, *Streptococcus salivarius*, *Eggerthella* spp., *Megasphaera elsdenii* ↓ *Dialister invisus*, *R. hominis*, *F. prausnitzii* **Functional pathways** ↑ Sugar phosphotransferase systems, ATP- binding cassette transporters of amino acids, bacterial secretion system, microcin C transport system, autoinducer-2 (AI-2) transport system ↓ Modules of V-type ATPase, pyruvate:ferredoxin oxidoreductase, bacterial ribosomal proteins	↑ *F. prausnitzii*, *Bacteroides caccae*, *Bacteroides finegoldii*, *Collinsella intestinalis* ↓ *E. coli*, *A. muciniphila*, *C. bartlettii* **Functional pathways** ↑ Modules of V-type ATPase, pyruvate:ferredoxin oxidoreductase, bacterial ribosomal proteins ↓ II–IV secretion system, AI-2 transport system
**Gravdal 2023 [82]**	↑ *Dorea* spp., *Pseudomonadota*, *Enterobacterales*, *Shigella* spp., *Escherichia* spp. ↓ *Roseburia* spp., *Roseburia intestinalis*, *Faecalibacterium prausnitzii*, *Dialister invisus*, *Veillonella* spp., *Sutterella wadsworthensis*	N/A
**Zhang 2021 [83]**	↑ *Pseudomonadota*, *Haemophilus*, *Escherichia/Shigella*, *Megasphaera* ↓ *Lachnospira*, *Paraprevotella*, *Helicobacter*	↑ *Negativicutes* ↓ *Finegoldia*, *Bacteroides*
**Diener 2020 [84]**	↑ *Escherichia*, *Veillonella* ↓ *Anaerostipes*, *Anaerostipes hardus*, *Blautia*	↑ *Escherichia*, *Veillonella* ↓ *Anaerostipes*, *Blautia*
**Allin 2018 [121]**	↑ *Dorea*, *[Ruminococcus]*, *Sutterella*, *Streptococcus* ↓ *Clostridium*, *Akkermansia muciniphila*	N/A
**Nuli 2019 [122]**	↓ α-diversity ↑*Bacillotta*, *Actinomycetota*, *Saccharibacteria*, *Megamonas*, *Haemophilus*, *norank_p_Saccharibacteria* ↓ *Bacteroidota*, *Pseudomonadota*, *Ruminococcaceae*, *Barnesiella*, *Sutterella*, *Ruminiclostridium*, *Eubacteriales*, *Coriobacteriaceae*, *Flavonifractor*	↑ *Deferribacteres*, *Moryella*, *Lachnospiraceae_NC2004_group*
**Ghaemi 2020 [123]**	↑ *E. coli*, *Bacteroides fragilis* ↓ *F. prausnitzii*	↓ *Bifidobacterium*

**Note:** Only the statistically significant results referring to T2D patients without antidiabetic or other treatment are presented. ↓: Decreased; ↑: Increased; NGT: Normal glucose tolerance; Pre-D: Pre-diabetes; T2D: Type 2 diabetes; richness: a simple count of species; β-diversity: the ratio between regional and local species diversity; α-diversity: the mean species diversity in sites or habitats at a local scale.; N/A: Non Applicable.

**Table 3:** The effect of exercise training on gut microbiota composition and function in pre-D and T2D.

Study	Study Group	Exercise Intervention	Changes in T2D Related Indices	Changes in GM Composition	Changes in GM Function
**Lambert 2015 [136]**	6-week-old mice: T2D db/db sedentary, db/db exercise; Non-D: db/+ sedentary, db/+ exercise	6 weeks of: (a) Moderate to high intensity treadmill running, 60–66 min/session, 5 days/week, or, (b) sedentary behavior		db/+ mice: *↑ Bifidobacterium* spp., *Lactobacillus* spp., and Clostridium leptum, Clostridium cluster I; ↓ *Bacteroides/Prevotella* spp., and *Methanobrevibacter* spp. db/db mice: ↓ Total bacteria and *Enterobacteriaceae*, *Bifidobacterium* spp., *Bacteroides/Prevotella* spp., and *Methanobrevibacter* spp., *Clostridium cluster I*; *↑ Lactobacillus* spp., and *C. leptum*	
**Pasini 2019 [137]**	30 male patients, with T2D for at least 2 years **Medication** Glucose, lipid, and blood pressure lowering agents	6 months of: Aerobic, resistance and flexibility training, 90 min/session, 3 times/week	↓ BW, BMI, fat mass, FPG, HOMA index, CRP, CHOL-T ↑ Lean body mass, VO_2max_	*↓ Candida albicans*, and *Mycetes* spp. **Intestinal barrier health** *↓ Zonulin* in feces	
**Motiani 2020 [138]**	26 sedentary insulin-resistant male/ female patients with pre-D and T2D **Medication** No medication (n = 4), Metformin (n = 11), DPP-IV (n = 5), sulfonylurea (n = 1)	2 weeks of: (a) Sprint interval training (SIT), 3 times/week, or, (b) Moderate intensity continuous training (MICT), 3 times/week	**T2D related and functional indices** SIT: *↑* VO_2peak_ SIT & MICT: *↓* % BF, abdominal visceral fat mass, HbA1c, TNF-α, and LBP **Intestinal fasting fatty acid uptake (IFAU) & glucose uptake (GU)** SIT: *↔* GU, IFAU MICT: *↔* IGU, FAU in the duodenum or colon; ↓ FAU in the jejunum	SIT & MICT: *↓ Bacillotta/Bacteroidota ratio*, *Blautia* spp., *Clostridium* spp.; *↑ Bacteroidota* SIT: *↑ Lachnospira*, *Bacteroides* MICT: *↑ Veillonella*, *Veillonella dispar*, *Faecalibacterium prausnitzii*	
**Torquati 2022 [139]**	12 T2D patients participating in <50 min/week of moderate intensity or 75 min/week of vigorous intensity physical activity **Medication** Metformin Or Metformin + DPP-4 inhibitors	8 weeks of: (a) High intensity combined aerobic and resistance training, 26 min/session, 3 sessions/week (C-HIIT), or (b) Moderate intensity combined aerobic and resistance training, 52 min/session, 4 sessions/week (C-MICT)	C-HIIT vs. C-MICT: *↔*FPG, HbA1c, CHO-T, HDL, LDL, TG, hs-CRP, SBP, DBP, BW, WC, VO_2peak_	C-MICT: ↑ *Bifidobacterium*, *C. leptum*, *F. prausnitzii*, *A. municiphila*, *Escherichia* spp., *Enterococcus* spp., *Lachnospira eligens*, *Agathobaculum* spp., *Massimaliae* spp. C-HIIT: ↑ *Erysipelotrichales*, *Methanobrevibacter smithii*, *Oscillospirales*, *Ruminococcus bromii*, *Negativibacillus* spp.	C-MICT: ↑ pathways for pyruvate metabolism (ko00620), AAs metabolism (ko00250, ko00260, ko00290) C-HIIT: ↓ pathways for bacterial secretion system—translocase/protein export SecD (K03072), Transcription regulation (Prokaryotic defense system K07316, Transcription factor K05499), Peptidase and inhibitors (K20608)
**Liu 2020 [140]**	34 sedentary male individuals with pre-D (IGT and/or IFG) **Medication:** No antidiabetic medication, no antibiotics, no probiotics	12 weeks of: (a) High intensity combined aerobic and strength training, 70 min/session, 3 sessions/week, or, (b) Sedentary behavior Responders and non-responders to exercise	Exercise group (whole group): ↓ Fasting INS, HOMA-IR, TG, CHO-T, resting HR, hs-CRP, BW, % BF Responders: ↓ 42.70% in fasting INS, 49.60% in HOMA-IR; ↑ 116.29% in Matsuda index Non-responders: *↔*	Responders: ↑ *Lachnospiraceae*, *Streptococcus mitis; * ↓ *Akkermansia muciniphila*, *Alistipes putredinis*, *Alistipes shahii*, *Bacteroides cellulosilyticus*, *Bacteroides clarus*, *Bacteroides xylanisolvens* Non-responders: ↑ *A. shahii * ↓ *Escherichia coli*, *Ruminococcus gnavus*	Responders: ↑pathways for quorum sensing, DNA replication, amino acid metabolism, glycan biosynthesis, lipid metabolism, BCAAs catabolism, SCFAs biosynthesis Non-responders: ↑ pathways for catabolism of AAAs and SAAs, production of colonic gases, and detrimental substrates for oxidative stress and inflammation

**Note**: ↑: Increased; ↓: Decreased; ↔: Similar between groups; Pre-D: Pre-diabetes; T2D: Type 2 diabetes; GM: Gut microbiota; BW: Body weight; BMI: Body mass index; FPG: Fasting plasma glucose; HOMA-IR: Homeostasis Model Assessment-Insulin Resistance; CRP: C-reactive protein; CHO-T: Total cholesterol; *VO_2max_*: Maximal oxygen consumption; DPP-IV: Dipeptidyl peptidase-4; %BF: Body fat percentage; HbA1c: Glycated hemoglobin; TNF-α: Tumor necrosis factor α; LBP: Lipopolysaccharides-binding protein; HDL: High-density lipoprotein; LDL: Low-density lipoprotein; TG: Triglycerides; hs-CRP: High-sensitivity CRP; SBP: Systolic blood pressure; DBP: Diastolic blood pressure; WC: Waist circumference; VO_2peak_: Peak oxygen consumption; IGT: Impaired glucose tolerance; IFG: Impaired fasting glucose; INS: Insulin; HR: Heart rate; BCAAs: Branched-chain amino acids; SCFAs: Short chain fatty acids; AAAs: Aromatic amino acids; SAAs: Sulfur-containing amino acids.
